# Integrative Genomics with Mediation Analysis in a Survival Context

**DOI:** 10.1155/2013/413783

**Published:** 2013-12-18

**Authors:** Szilárd Nemes, Toshima Z. Parris, Anna Danielsson, Zakaria Einbeigi, Gunnar Steineck, Junmei Miao Jonasson, Khalil Helou

**Affiliations:** ^1^Division of Clinical Cancer Epidemiology, Department of Oncology, Institute of Clinical Sciences, Sahlgrenska Academy at University of Gothenburg, Gothenburg, Sweden; ^2^Department of Oncology, Institute of Clinical Sciences, Sahlgrenska Academy at University of Gothenburg, Gothenburg, Sweden; ^3^Division of Clinical Cancer Epidemiology, Department of Oncology and Pathology, Karolinska Institutet, Stockholm, Sweden

## Abstract

DNA copy number aberrations (DCNA) and subsequent altered gene expression profiles may have a major impact on tumor initiation, on development, and eventually on recurrence and cancer-specific mortality. However, most methods employed in integrative genomic analysis of the two biological levels, DNA and RNA, do not consider survival time. In the present note, we propose the adoption of a survival analysis-based framework for the integrative analysis of DCNA and mRNA levels to reveal their implication on patient clinical outcome with the prerequisite that the effect of DCNA on survival is mediated by mRNA levels. The specific aim of the paper is to offer a feasible framework to test the DCNA-mRNA-survival pathway. We provide statistical inference algorithms for mediation based on asymptotic results. Furthermore, we illustrate the applicability of the method in an integrative genomic analysis setting by using a breast cancer data set consisting of 141 invasive breast tumors. In addition, we provide implementation in R.

## 1. Introduction

Concomitant analysis of the two biological levels, DNA and RNA, and elucidating their implication in cancer development and cancer-related mortality is a key objective of studies within the cancer genetics field. Integrative analysis of DNA copy number aberrations (DCNA) and mRNA levels has received considerable interest with studies employing a wide range of statistical methods [1–5]. Integrative genomic analyses aim to identify novel biomarkers that can distinguish between patients with favorable and unfavorable prognosis. However, the sole focus on DCNA-driven altered gene expression profiles falls short of this goal. To develop a better understanding of the impact DCNA-driven altered gene expression profiles have on tumor recurrence or cancer-specific mortality, we need to consider patient survival status and survival time, that is, survival analysis. However, the unique relationship between DNA and RNA raises the need to interpret the data from a more refined viewpoint. Ascertaining causality between two biological factors is far to be straightforward. However, no one would question that mRNA is transcribed from a DNA template. Thus, mRNA mediates the genetic information imprinted in DNA and possibly the effect copy number aberrations have on survival status. The chosen statistical-mathematical framework has to properly address this issue. Mediation assumes that an independent variable (DNA) causes the mediator (mRNA), which in turn causes the outcome (survival status). Thus, the mediator accounts partially or totally for the relation between the predictor and outcome. The concept and methodology of mediation were recently extended to survival analysis [6–8], but so far forays into genetics are limited or nonexistent. Genomic data poses specific challenges to an analyst [9]. The most obvious challenge is the need of testing thousands of markers simultaneously that raises the need of an automatized procedure. In this note, we illustrate a framework to apply integrative genomic analysis of DNA copy number aberrations and mRNA levels in a mediation analysis context to test DNA copy number-mRNA-survival pathways. If a mediation effect is observed for a specific gene, we hypothesize that the specific gene is a driver gene. If no mediation effect is observed then possible associations between DNA copy number aberration and the outcome are likely to indicate passenger genes. Naturally, other factors than DNA copy number aberration influence gene expression and mRNA levels; thus, a gene can be causally linked with the outcome without any apparent association between copy number aberrations and survival. Our specific aim is to provide Delta-method-based asymptotic statistical inference for the mediation effect feasible to test large numbers of probes simultaneously. We examine the properties of the advocated approach with the help of a simulation study and illustrate applicability using genomic and transcriptomic microarray data from 141 invasive breast tumors.

## 2. Methods

### 2.1. Data

Through the whole paper we assume that the data consist of DNA copy number aberration data, messenger RNA data for every patient, and the associated survival length and status. Moreover, we assume that the date is kept on their original log_2_ ratio scale. These values represent the logarithm of the normalized copy number and mRNA measurements with samples from healthy tissues. The logarithm has base 2. Both DCNA and mRNA values generally were between −2 and 2 on a continuous scale. A reading of 0 indicates equal amounts of genetic material in tumor and normal tissues; any value above 0 indicates gains of DNA in tumor cells (a value of 1 indicated double amounts of DNA in tumors compared with normal tissues). Inversely, negative values indicate loss of genetic material (a value of −1 indicates a heterozygous loss of genetic material compared with normal tissues). Naturally, other data types can be used as well, but we advise the readers to keep measurements on continuous scale and do not discretize the data (e.g., loss/normal/gain).

### 2.2. The Model for the Mediation

**Figure 1:**
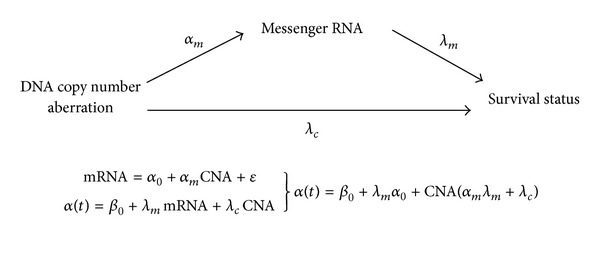
Schematic representation for the mediation of DNA copy number aberrations effect on survival by messenger RNA. The *α*_*m*_ represents the change in messenger RNA levels as a result of one unit change in DNA copy number aberrations as modeled by a linear least squares regression. The *λ*_*m*_ and *λ*_*c*_ represent the regression coefficients for Aalen's' additive hazard model when the survival status is regressed on messenger RNA and DNA copy number aberration levels, respectively.

Mediation occurs when the effect of one variable, the antecedent (DNA copy number aberration) on the outcome, is transmitted through a mediating variable (mRNA). Mediation explicitly assumes the existence of a causal chain, and it can be modeled by a series of regression equations. The mediator model for the DNA copy number-mRNA-survival pathway is illustrated in Figure 1. The parameters of the mediator model are *α*_*m*_, the effect of DNA copy number aberration on mRNA levels; *λ*_*m*_ the effect of mRNA on survival; and *λ*_*c*_ the direct effect of DNA copy number aberration on the survival status. These later two parameters are estimated by Aalen's Additive Hazards model, a flexible nonparametric regression [10, 11] while *α*_*m*_ is estimated by an ordinary least squares regression. Of immediate interest is the product of *α*_*m*_ and *λ*_*m*_ that constitutes the estimator for the mediation effect, *α*_*m*_ *λ*_*m*_. Moreover, beside mediation one could be interested in testing the total effect (*α*_*m*_ *λ*_*m*_ + *λ*_*c*_) or ratio between the mediated and total effect, the relative magnitude, *P*_*M*_, that gives an informative gauge of the relevance of the mediated effect compared to the total effect of the covariate and is estimated as

(1) $$\begin{array}{c} P_{M}=\frac{\alpha_{m}\lambda_{m}}{\alpha_{m}\lambda_{m}+\lambda_{c}}. \end{array}$$

Transcription is the first step of gene expression, in which a particular segment of DNA is copied into RNA by the enzyme, RNA polymerase. This step represents the first equation in the mediation analysis, namely, the effect of copy number aberrations on mRNA. The assumption is that the number of copies of a gene available in the cells has a direct effect on the amount of mRNA molecules synthetized. The second step in the mediation process is the translation, when messenger RNA (mRNA) is decoded by a ribosome complex to produce a specific amino acid chain, or polypeptide, eventually an active protein that ultimately affects tissue and organ functioning, thus survival.

### 2.3. Statistical Inference for the Mediation and Total Effect

Statistical inferences for the parameters of the mediator model are *α*_*m*_, *λ*_*m*_, and *λ*_*c*_ is straightforward based on the central limit theorem for *α*_*m*_ and on the martingale central limit theorem for *λ*_*m*_ and *λ*_*c*_. The null hypothesis usually states that the parameters are equal to zero (no effect) while the alternative states that these parameters are not zero. Similarly, we can test if the mediation effect is *α*_*m*_ *λ*_*m*_ = 0; if we see evidence *α*_*m*_ *λ*_*m*_ ≠ 0 then we can establish mediation. This can be easily translated to a null-hypothesis framework *H*_0_ : *α*_*m*_ *λ*_*m*_ = 0 against the alternative *H*_1_ : *α*_*m*_ *λ*_*m*_ ≠ 0. For this is necessary to know the distribution of the mediation estimate. Both *α*_*m*_ and *λ*_*m*_ are unbiased and normally distributed; however their product is not normally distributed [12, 13], but it follows the normal-product distribution; a leptokurtic distribution that is symmetrical around is mean [14]. While the derivation of the distribution of products of random variables is straightforward, implementation is rather difficult [15]. Similarly, we could be interested in the total effect testing the null-hypothesis of *H*_0_ : *α*_*m*_ *λ*_*m*_ + *λ*_*c*_ = 0 against the alternative *H*_1_ : *α*_*m*_ *λ*_*m*_ + *λ*_*c*_ ≠ 0. Additionally, it is desirable to build a confidence interval for *P*_*M*_. For either the total effect or relative magnitude there are no routines for the derivation of the limiting distribution.

Clearly we need alternative routines, such as computer intensive methods (e.g., bootstrap and permutations) or approximations. The Delta method is a method for deriving an approximate probability distribution for a function of an asymptotically normal statistical estimator [16].

Application of the Delta method leads to the following variance estimator for the mediation parameter:

(2) $$\begin{array}{c} \sigma_{\text{Med}}^{2}=\alpha_{m}^{2}\sigma_{\lambda_{m}}^{2}+\lambda_{m}^{2}\sigma_{\alpha_{m}}^{2} \end{array}$$

for the total effect of

(3) $$\begin{array}{c} \sigma_{\text{Tot}}^{2}=\sigma_{\lambda_{c}}^{2}+\alpha_{m}^{2}\sigma_{\lambda_{m}}^{2}+\lambda_{m}^{2}\sigma_{\alpha_{m}}^{2}+2\alpha_{m}\sigma_{\lambda_{m}\lambda_{c}} \end{array}$$

and for the relative magnitude which takes a more complicated form and is given by

(4) σPM2=(λmαm(αmλm+λc)2)2σλc2 +(αm(αmλm+λc)−λmαm2(αmλm+λc)2)2σλm2 +(λm(αmλm+λc)−λm2αm(αmλm+λc)2)2σαm2 +(αmλm)(αm(αmλm+λc)−λmαm2)(αmλm+λc)4σλcλm2.

For the technical details we refer the reader to Supplementary Material available online at http://dx.doi.org/10.1155/2013/413783.

Statistical inference is straightforward, using the derived variance estimators and parameter estimates from standard statistical software. Both confidence intervals and *P* values are easy to calculate based on the approximately normal distribution of the estimates.

Approximate confidence interval for mediation effect is calculated as (*α*_*m*_ *λ*_*m*_ − *Z*_*α*/2_ *σ*_Med_; *α*_*m*_ *λ*_*m*_ + *Z*_*α*/2_ *σ*_Med_). The procedure is similar for the total effect and ratio, just with the suitable changes. Confidence intervals for the direct effect can be obtained in a similar way based on the output of the Aalen's additive model.

As it is not straightforward how to adjust confidence intervals for multiple testing, we need to calculate *P* values. We test the null hypothesis of no effect *H*_0_ : *α*_*m*_ *λ*_*m*_ = 0 against the alternative *H*_1_ : *α*_*m*_ *λ*_*m*_ ≠ 0. Based on the approximately normal distribution of the estimates we can calculate a test statistics, *Z*-score as *α*_*m*_ *λ*_*m*_/*σ*_Med_, with *Z* ~ *N*(0,1). Inference for the total effect proceeds with the same steps.

Alternatively statistical inference for the mediated effect, but not the total effect, can be obtained by the means of product normal distribution [14, 17]. Bootstrapping or other computer intensive methods are easy and straightforward alternates; however their applicability is limited by the large number of genes that integrative genomic studies routinely consider.

### 2.4. Simulation Study

To investigate the properties of the proposed testing procedure with sample sizes attainable in real life studies, we conducted a series of simulation studies. The simulation studies were designed to offer insight into the efficiency of the estimator for the mediated total effect. Moreover, we considered the adequacy of 95% confidence intervals.

Based on a previous analysis on 97 tumors we estimated the mean log_2_ ratio values for DNA copy number aberrations at *μ* = 0.248 and *σ* ^2^ = 0.047. Thus, we simulated the DNA copy number data as normally distributed with *μ* = 0.248 and *σ* ^2^ = 0.047. Furthermore, based on the same data we generated the relative mRNA log_2_ ratio values by −0.578 + 0.775DCNA + *ε* with *ε* ~ *N*(0,0.158). Survival times were generated according to the additive hazard model with *α*(*t* | **x**_*i*_) = *β*_0_ + *λ*_*m*_mRNA + *λ*_*c*_DCNA, where *λ*_*m*_ = 0.4 and *λ*_*c*_ = 0.1, leading to an indirect effect of 0.31 and a total effect of 0.41. The baseline *β*_0_ was set to 1 and censoring was chosen to 0.9 to obtain a censoring around 60%, relevant for cancer studies. We created data sets with samples sizes from 25 to 300 with increment of 25. At each sample size we created 1000 samples.

#### 2.4.1. Properties of the Estimator

As unbiased and accurate estimates are the foundation of a proper significance testing we estimated the bias and accuracy of the mediation effect estimator at varying sample sizes. We calculated bias as $\delta =\overset{-}{\hat{\beta }}-\beta$ and estimated accuracy with the help of mean squared error $\text{MSE}={\left(\overset{-}{\hat{\beta }}-\beta \right)}^{2}+{\left(\text{se}\left(\hat{\beta }\right)\right)}^{2}$.

#### 2.4.2. Confidence Interval Adequacy

As a first gauge of confidence interval adequacy we used the coverage level, defined as the proportion of times the obtained confidence interval contains the true, specified parameter. The coverage expectedly should be close to the chosen nominal level, for example, 95%. Over-coverage indicates too conservative confidence intervals and low statistical power, while under-coverage indicates overconfidence and it will result in false positive findings [18]. However, we cannot expect that the coverage of a proper confidence interval to be equal to the chosen nominal level. Rather due to the randomness and limited number of simulations, we would expect that the coverage will situate close to the nominal level and in-between tolerance limits whose boundaries are determined by the number of simulations performed. If we regard one confidence interval then we have a clear dichotomy: the confidence interval covers or not the true, specified parameter. This follows the Bernoulli distribution, and if we consider all confidence intervals constructed the Binomial distribution. Thus, for a 95% confidence interval we expect that the coverage should not fall outside of approximately 1.96 standard errors of the coverage probability (*p*), $p\pm 1.96\sqrt{1\left(1-p\right)/N}$, where *N* is the number of simulations. As we run 1000 simulation per sample size we expect that the coverage should be between 0.936 and 0.963. Any other value indicates over- or under-coverage.

A second gauge of confidence interval adequacy was the its width, methods that provide confidence intervals with adequate coverage and narrower confidence intervals provide the best trade-off between type I and type II errors.

Thirdly, we would expect that the 95% constructed confidence intervals to fall below the true value about 2.5% times and above equally often [19]. Thus, if we run 1000 simulations we expect that 25 times (lower error) the confidence intervals fall below the true parameter and 25 times above (upper error), deviations from this indicating lack of symmetry.

Additionally we compared the performance of the proposed approach to inferential procedures based on normal product distribution and nonparametric bootstrap confidence intervals (normal, base, percentile, and bias corrected and accelerated).

### 2.5. Application on Breast Tumors

Primary invasive tumors (*n* = 141) from 141 breast cancer patients were selected from the fresh-frozen tissue tumor bank at the Sahlgrenska University Hospital Oncology Lab (Gothenburg, Sweden). All samples were assessed for DNA content at the time of diagnosis from 1991 to 1999 (data not shown) by flow cytometry at the Laboratory for Clinical Chemistry, Sahlgrenska University Hospital. The presence of malignant cells was assessed in all samples by evaluation of touch preparation imprints stained with May-Grünwald Giemsa (Chemicon). All procedures were done in accordance with the Declaration of Helsinki and approved by the Medical Faculty Research Ethics Committee (Gothenburg, Sweden).

Whole-genome tiling arrays with 38,043 reporters mapping to the UCSC May 2004 hg17: NCBI Build 35 were manufactured as previously described [20] at the SCIBLU Genomics DNA Microarray Resource Center (SCIBLU), Department of Oncology, Lund University. Images and raw signal intensities were acquired using an Agilent G2505B DNA microarray scanner (Agilent Technologies) and GenePix Pro 6.0.1.22 (Axon Instruments) image analysis software. Data preprocessing and normalization were done using the web-based BioArray Software Environment system (BASE) provided by SCIBLU (http://base2.thep.lu.se/onk/).

The RNA samples were processed at SCIBLU using Illumina HumanHT-12 Whole-Genome Expression BeadChips (Illumina), according to the manufacturer's instructions. The expression microarrays contained approximately 49,000 probes representing >25,400 RefSeq (Build 36.2, Release 22) and Unigene (Build 199) annotated genes. Images and raw signal intensities were acquired using the Illumina BeadArray Reader scanner and BeadScan 3.5.31.17122 (Illumina) image analysis software, respectively.

Data preprocessing and quantile normalization were applied to the raw signal intensities using BASE. Further data processing was done in Nexus Expression 2.0 (BioDiscovery) using log_2_-transformed, normalized expression values and a variance filter. Normalized values from five normal breast samples profiled with Illumina HumanWG-6 Expression BeadChips (GEO, accession number GSE17072) were used as reference [21]. Further details the reader will find in Parris et al. [22] and its supplementary material. The individual array-CGH and expression microarray data are accessible through the National Center for Biotechnology Information (NCBI) Gene Expression Omnibus (GEO accession numbers GSE20486 and GSM304824).

The data from the two genomic platforms were matched [23]. In conclusion out data set consist of 141 breast tumours belonging to 141 breast cancer patients and from each tumour 8349 genes were analysed. These data were selected for illustrative purposes.

Adjustment for multiple testing was done with method of Benjamini and Hochberg to control the false discovery rate.

## 3. Results

### 3.1. Properties of the Estimator

**Figure 2:**
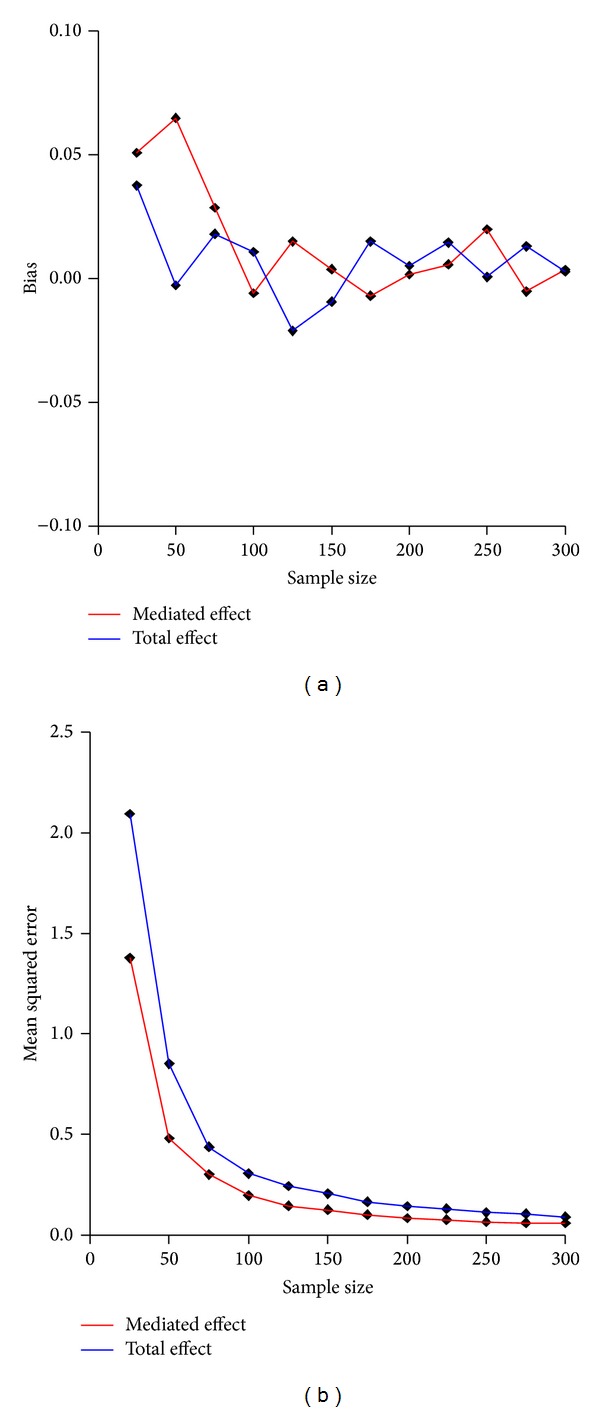
Bias and mean squared error of the estimated mediated and total effects.

**Figure 3:**
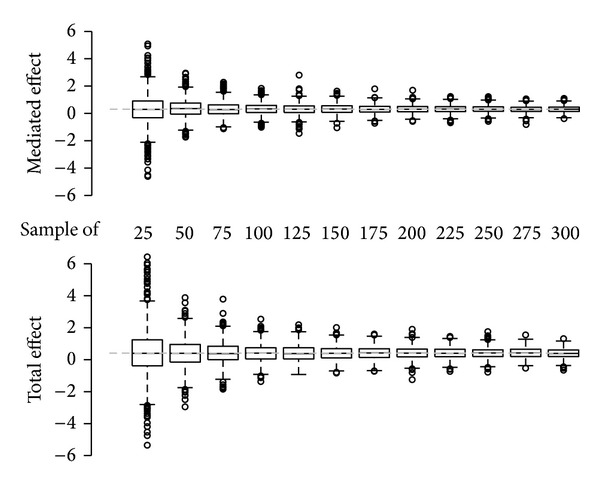
Sample size dependent distribution of the estimated mediated and total effects.

At sample sizes under 50 the estimator proved to overestimate systematically the mediated and total effects (Figure 2). Moreover the mean squared error at small sizes was considerably higher than the estimated effects itself. This partially was due to biased estimates but also due to the high variability observed at small sample sizes (Figure 3).

### 3.2. Properties of the Confidence Interval

**Figure 4:**
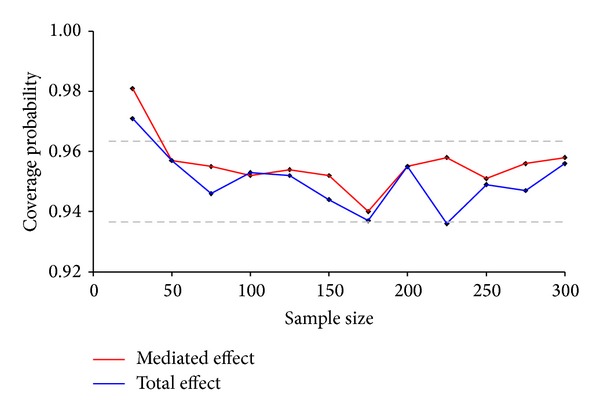
Sample size related coverage probabilities for the 95% Delta-method based confidence intervalfor the mediated and total effects. The dashed grey line represents the acceptance limits for 1000 simulations.

The coverage of the 95% confidence interval varied considerably with sample size (Figure 4). At small samples the Delta-method based confidence intervals systematically exceeded the nominal level. This was characteristic even for confidence intervals based on normal and base bootstrapping (Table 1). At sample sizes over 100 the coverage level was close to the nominal value and was in between the acceptance limits. Similarly at sample sizes above 100 the comparative analysis showed that the 95% confidence interval based on the Delta-method had similar or slightly better coverage and width than confidence intervals based on nonparametric bootstrapping. Similarly, the coverage and width coincide well with the confidence intervals based on the normal product distribution (Table 1). Moreover, the confidence interval based on the Delta-method was symmetrical; intervals that failed to cover the true population value fell roughly equal to the lower and upper tail of the distribution.

### 3.3. Analysis of Breast Tumors

Analysis of 8,349 chromosome segments spread over the entire genome revealed that DCNA explains observed mRNA levels for 2,790 genes (Supplementary Figure 1). mRNA levels showed significant association with survival for 288 genes, but only 128 genes showed significant DCNA-mRNA and subsequent mRNA-survival association. After adjusting for multiple testing, none of these 8,349 genes showed a significant mediation effect. If we only tested the 128 genes with both DCNA-mRNA and mRNA-survival association, then all 128 genes showed significant mediation effects. Among these 128 genes, the mRNA levels for 124 genes mediated completely the effect of DCNAs on survival and no significant direct effect of DCNAs on survival was recorded. For four fragments we observed that mRNA levels exhibited significant mediation effect but DCNAs exerted an effect on survival that was not mediated by mRNA levels belonging to that specific fragment.

## 4. Discussion

In the present study, we proposed a novel approach to integrate DNA copy number aberrations and gene expression patterns (mRNA levels) with survival that might offer insights into the biological mechanisms of cancer-related mortality. We specifically aimed to provide a methodology applicable to large scale genomic studies that is easy to implement and interpret.

The advocated approach is largely based on and expands upon the current advances in mediation analysis in a survival context [7, 24–26] and adheres to the effort to infer causal association between genes and disease [27–31]. Ferkingstad et al. [32] offers an early example of this approach, though their approach is more practical in addressing the effect of target genes on survival. The novelty of the paper lies in the adaption of the mediation analysis to an integrative genomic setting and in providing a simple and feasible procedure to test the mediation effects concomitantly on a large number of probes. Our simulation results showed that the proposed inference based on the Delta-method is equivalent to inferential procedures based on normal product distribution or resampling, the staple methods of inference in mediation studies [33] in moderate and large samples. Beside lower computational load and shorter running time, the main advantage over resampling or simulation-based inference lies in the possibility of multiple adjustments. Multiple adjustments require *P* values with a resolution that makes resampling or simulation based inference unfeasible for integrative genomic studies. Moreover, the Delta-method based inference can be expanded to more complicated causal diagrams. The Distribution of Product estimator [17, 34] is a natural alternative to the Delta-method based inference with superior properties in small samples. However, is not clear how it can be extended to total effects, relative magnitude, or more complicated causal networks.

The simulation study that we performed was limited its scope however highlighted interesting aspects. Mainly, that extreme caution is advised at sample sizes under 100.

Premeditatedly, we omitted evaluating the relative magnitude, the ratio between indirect and total effect. However, we do present formulae of the variance. The relative magnitude, PM, prevails in the literature despite lack of straightforward interpretation [35]. Additionally, point and variance estimates for the relative magnitude are unstable and require extreme sample sizes for a stable point estimation and correct inference [36, 37].

In this note, we have focused on the statistical and practical issues, and by using a breast cancer data set illustrate the applicability of the method. The occurrence and progression of neoplastic disease require multiple genetic events, ranging from single nucleotide mutations to large DNA regional rearrangements, occurring sequentially in a cell lineage. These genetic events have major effects on the process of mRNA transcription and further protein translation. This small scale study showed that around 36% of DCNA changes are mirrored on the mRNA level and the remaining 64% could be a result of genomic instability of the analyzed carcinomas (i.e., passenger genes). However, from the 8,349 DNA fragments that we considered initially, we recorded no mediation effect. This could have both biological and mathematical reasons. One has to recall that both the DCNA and mRNA data originate from tumors that were surgically removed. Thus, one could expect that their effect diminishes as time passes. Though, striking genomic similarities between primary and secondary tumors that develop often years apart [38, 39] indicate their implication, not only in the cancerous process but in patient survival times. Beyond the biological reasoning, sample size issues common for genomic studies persists. The relatively small sample and gene ration in our sample (141 patients and 8,349 segments) imposed a rather stringent multiple testing threshold. Mediation analysis is common in psychology and social sciences were generally one (or perhaps a few) mediation analysis per study is performed. Thus, there was no immediate need for adjustment for multiple testing. Fritz and MacKinnon [40] concluded that generally a sample size larger than 400 is desired to achieve adequate statistical power. The proper multiple adjustment strategy for mediation analyses is yet to be determined. Likely, a prefiltering of the data based on biological clinical reasoning is needed (e.g., consider only genes with above a predefined copy number change). However, as drawing biological conclusions was beyond the scope of our work we did not consider any specific pre-filtering of the data in order not to over-optimize the data to the method [41].

An attempted filtering based to the assumptions of mediation (the independent variable (DNA) causes the mediator (mRNA) which in turn causes the outcome) reduced the data to only 128 genes located in 128 DNA segments. All the 128 genes in this reduced sample showed significant mediation. For 124 of these 128 segments, mRNA seemingly mediated the entire effect that DCNA had on survival. These segments likely contain oncogenes or tumor suppressor genes involved in tumor initiation and progression. For the remaining 4 genes, the effect of DCNA was not solely mediated by mRNA produced within the fragment. These segments may contain sequences with regulatory elements or encode microRNA with transacting effects. Conversely, expression of these genes could be affected by transacting genes with global effect (e.g., master predictors as coined by Peng et al. [42]).

As this small example illustrated considering mediation could highlight interesting aspects that classical integrative genomic or survival analysis would miss. Classical integrative genomics considers association between DCNA and mRNA and aims to assess the strength of association between the two biological levels without any clinical endpoint. Classical survival analysis (e.g., Cox regression) would also miss important aspects. A Cox regression model describing the effect of DCNA and mRNA on survival will consider the two as independent factors. If the effect of DCNA on survival is mediated by mRNA and there is no direct effect, a Cox regression model will miss this effect and it will conclude that DCNA has no effect on survival status. Moreover, in opposition to the Aalen's additive model decomposition of Cox regression estimates into direct and mediated effects lack any straightforward analytical expression and there are no general measures for a mediated effect [6].

The expansion proposed in this paper not only adds a clinically relevant endpoint to the two biological levels but also builds a biologically plausible model and offers ways of inference. Naturally, this adds an extra layer of complexity to an already complex issue, but we believe the gains outweigh the impediments.

Here, we merely focus on integrative genomic studies while the method can easily be applied to other study types as well. Similarly, generalizations for more than one mediator or more than one pathway are straightforward. Researchers could test models based on known pathways or empirical models resulting from large scale network modeling [43] built on the premise that DCNA can have global effects beside effects on those genes contained within that DCNA [44]. Moreover, causal networks incorporating covariates other than genomic data can be considered. The structure of the causal diagram and minimal sufficient adjustment sets for estimating mediated (or) total effects can be determined using appropriate epidemiological theory and tools [45].

## 5. Conclusions

We believe that mediation analysis can be a useful addition to the toolbox of bioinformaticians and geneticists seeking to integrate DNA copy number aberrations, altered gene expression profiles, and patient survival.

## Supplementary Material

This Supplementary Materials contains the theoretical background for the Delta-method and a brief derivation of the variance estimators. Moreover, we provide the variance estimators for the extended casual path when the hypothesized effect of DNA copy number aberrations on survival is mediated by the mRNA-protein pathway. The Supplementary Materials concludes with a short R script that implements the advocated method.

## Figures and Tables

**Table 1:** Results of simulation of the characteristics of the 95% CI for the mediation effect. The lower and upper errors denote the number of times when these intervals fell below or above the true population value.

	Coverage	Lower error	Upper error	Width
*n* = 25				
Delta method	0.977	0.003	0.020	4.288
Product normal	0.951	0.015	0.034	4.514
Boot				
Normal	0.995	0.001	0.004	6.153
Base	0.996	0.001	0.003	6.286
Percentile	0.944	0.029	0.027	6.286
BCa	0.952	0.024	0.024	6.224

*n* = 50				
Delta method	0.971	0.006	0.023	2.587
Product normal	0.954	0.018	0.028	2.663
Boot				
Normal	0.984	0.004	0.012	3.036
Base	0.987	0.003	0.010	3.118
Percentile	0.942	0.031	0.027	3.118
BCa	0.954	0.022	0.024	3.099

*n* = 75				
Delta method	0.969	0.009	0.022	2.008
Product normal	0.961	0.015	0.024	2.047
Boot				
Normal	0.980	0.007	0.013	2.215
Base	0.985	0.004	0.011	2.268
Percentile	0.950	0.027	0.023	2.268
BCa	0.963	0.020	0.017	2.253

*n* = 100				
Delta method	0.960	0.015	0.025	1.672
Product normal	0.947	0.025	0.028	1.696
Boot				
Normal	0.965	0.012	0.023	1.801
Base	0.977	0.007	0.016	1.839
Percentile	0.943	0.027	0.030	1.839
BCa	0.947	0.025	0.028	1.831
