# Supplementary material for: Integrative Genomics with Mediation Analysis in a Survival Context
Source: Comput Math Methods Med. 2013 Dec 18;2013:413783. doi: 10.1155/2013/413783 (PMC3878392; doi:10.1155/2013/413783)
Supplement: Supplementary file 1 — This Supplementary Materials contains the theoretical background for the Delta-method and a brief derivation of the variance estimators. Moreover, we provide the variance estimators for the extended casual path when the hypothesized effect of DNA copy number aberrations on survival is mediated by the mRNA-protein pathway. The Supplementary Materials concludes with a short R script that implements the advocated method. [file 413783.f1.pdf]

Supplementary materials

1. Statistical Inference

a. Delta-method based approximate inference

The Delta method is a method for deriving an approximate probability distribution for a function of an asymptotically normal statistical estimator from knowledge of the limiting variance of that estimator. If $\sqrt{n}\left(x-\mu_x\right)\xrightarrow{L} N(0,\sigma_x^2)$ then for a given function $f(x)$ with existing first-order derivative $\sqrt{n}[f(x)-f(\mu_x)]\xrightarrow{L} N(0,\sigma_x^2[f'(\mu_x)]^2)$, assuming that $f'(\mu_x)$ exists and it is non-zero [1]. The Delta-method applies a Taylor expansion to linearize a non-linear relationship. If a function $f(x)$ has derivatives of order k, then for a constant a the Taylor series of order k about a is

$$T_n(x)=\sum_{j=0}^{k}\frac{f^{(j)}(a)}{j!}(x-a)^j \ .$$

Generally, statistical literature and practical applications are interested mainly of the first order Taylor expansion and in a lesser extent in the second order expansion.

A second order expansion of $f(x)$ around μ_x gives

$$f(x)=f(\mu_x)+f'(x-\mu_x)+\frac{1}{2}f''(x-\mu_x)^2+R_{j\geq 3}(x)$$

where the reminder $R_{j\geq 3}(x)=(x-\mu_x)^j f^{(j)}(\xi)/j!$with $\xi\in(x,\mu_x)$ rapidly converges to zero. Following the notation of Preacher et al [2] we define the following parameters

- $\hat{\boldsymbol{\theta}}$ a column vector of regression coefficients used in the estimation of the mediated effect
- $\boldsymbol{\mu}_{\boldsymbol{\theta}}$ the expected values of the regression coefficients, $\boldsymbol{\mu}_{\boldsymbol{\theta}}=E\left[\hat{\boldsymbol{\theta}}\right]$
- $f\left(\hat{\boldsymbol{\theta}}\right)$ the effect of interest, the estimator for the mediation effect
- $\hat{\boldsymbol{\Sigma}}(\hat{\boldsymbol{\theta}})$ the estimated covariance matrix of $\hat{\boldsymbol{\theta}}$
- $\mathbf{D}=\partial_\theta f\left(\hat{\boldsymbol{\theta}}\right)$ the first order derivatives of $f\left(\hat{\boldsymbol{\theta}}\right)$evaluated at $\boldsymbol{\mu}_{\boldsymbol{\theta}}$, the Jacobian matrix of $f\left(\hat{\boldsymbol{\theta}}\right)$
- $\mathbf{H}=\partial_\theta^2 f\left(\hat{\boldsymbol{\theta}}\right)$ the Hessian matrix of $f\left(\hat{\boldsymbol{\theta}}\right)$evaluated at $\boldsymbol{\mu}_{\boldsymbol{\theta}}$

The Delta-method based variance is defined as

$$Var[f(\hat{\boldsymbol{\theta}})]\approx E\left[f(\hat{\boldsymbol{\theta}})^2\right]-\left(E\left[f(\hat{\boldsymbol{\theta}})\right]\right)^2 .$$

By the Taylor theorem we have

$$f\left(\hat{\boldsymbol{\theta}}\right) \approx f\left(\boldsymbol{\mu}_{\boldsymbol{\theta}}\right) + \left(\hat{\boldsymbol{\theta}} - \boldsymbol{\mu}_{\boldsymbol{\theta}}\right)\mathbf{D} + \frac{1}{2}\left(\hat{\boldsymbol{\theta}} - \boldsymbol{\mu}_{\boldsymbol{\theta}}\right)^2 \mathbf{H}$$

Without explicitly going through the algebra we give

$$E\left[f(\hat{\boldsymbol{\theta}})^2 \right] = f^2\left(\boldsymbol{\mu}_{\boldsymbol{\theta}}\right) + \mathbf{D}^T \hat{\boldsymbol{\Sigma}}(\hat{\boldsymbol{\theta}})\mathbf{D} + \frac{1}{4}\left(tr(\mathbf{H}\hat{\boldsymbol{\Sigma}}(\hat{\boldsymbol{\theta}}))\right)^2$$
$$+ \frac{1}{2}\left(tr(\mathbf{H}\hat{\boldsymbol{\Sigma}}(\hat{\boldsymbol{\theta}}))^2\right) + f\left(\boldsymbol{\mu}_{\boldsymbol{\theta}}\right) tr(\mathbf{H}\hat{\boldsymbol{\Sigma}}(\hat{\boldsymbol{\theta}}))$$

and

$$E\left[f(\hat{\boldsymbol{\theta}})^2 \right] = f^2\left(\boldsymbol{\mu}_{\boldsymbol{\theta}}\right) + f^2\left(\boldsymbol{\mu}_{\boldsymbol{\theta}}\right) tr\left(\mathbf{H}\hat{\boldsymbol{\Sigma}}(\hat{\boldsymbol{\theta}})\right) + \frac{1}{4}\left\{ tr\left(\mathbf{H}\hat{\boldsymbol{\Sigma}}(\hat{\boldsymbol{\theta}})\right)\right\}^2,$$

consequently $Var\left(f(\hat{\boldsymbol{\theta}})\right) = \mathbf{D}^T \hat{\boldsymbol{\Sigma}}(\hat{\boldsymbol{\theta}})\mathbf{D} + \frac{1}{2} tr\left\{\left(\mathbf{H}\hat{\boldsymbol{\Sigma}}(\hat{\boldsymbol{\theta}})\right)^2\right\}$

Variance estimator and inference for the mediated effect

As noted above $\alpha_m \lambda_m$ is the effect of DNA copy number aberrations on survival status mediated through mRNA, while $\alpha_m \lambda_m + \lambda_c$ is the total effect of DNA copy number aberrations on survival status. Using the above outlined notation we have that $\hat{\boldsymbol{\theta}} = \left[\hat{\alpha}_m, \hat{\lambda}_m\right]^T$ and $\boldsymbol{\mu}_{\boldsymbol{\theta}} = \left[\alpha_m, \lambda_m\right]^T$ and $f\left(\hat{\boldsymbol{\theta}}\right) = \hat{\alpha}_m \hat{\lambda}_m$. The gradient matrix of $f\left(\hat{\boldsymbol{\theta}}\right)$ is $\mathbf{D} = \partial_\theta f\left(\hat{\boldsymbol{\theta}}\right)\Big|_\mu$ and the Hessian matrix equals to

$$\mathbf{H} = \left.\begin{pmatrix} \partial^2_{\alpha_m\alpha_m} f\left(\hat{\boldsymbol{\theta}}\right) & \partial^2_{\alpha_m\lambda_m} f\left(\hat{\boldsymbol{\theta}}\right) \\ \partial^2_{\alpha_m\lambda_m} f\left(\hat{\boldsymbol{\theta}}\right) & \partial^2_{\lambda_m\lambda_m} f\left(\hat{\boldsymbol{\theta}}\right) \end{pmatrix}\right|_\mu .$$

As α_m and λ_m are independent from each other the estimator for the covariance matrix is

$$\boldsymbol{\Sigma}(\boldsymbol{\theta}) = \begin{pmatrix} \sigma^2_{\alpha_m} & 0 \\ 0 & \sigma^2_{\lambda_m} \end{pmatrix} \text{ while the Hessian, } \mathbf{H} = \begin{pmatrix} 0 & 1 \\ 1 & 0 \end{pmatrix}.$$

Plugging in the estimators for the mediation effect into the algorithm of the Delta method leads to the following variance estimator for the mediation parameter

$$\sigma^2_{Med} = \mathbf{D}^T\hat{\mathbf{\Sigma}}(\hat{\boldsymbol{\theta}})\mathbf{D} + \frac{1}{2}tr\left\{\left(\mathbf{H}\hat{\mathbf{\Sigma}}(\hat{\boldsymbol{\theta}})\right)^2\right\}$$
$$= \underbrace{\alpha_m^2\sigma^2_{\lambda_m} + \lambda_m^2\sigma^2_{\alpha_m}}_{first-order} + \underbrace{\sigma^2_{\lambda_m}\sigma^2_{\alpha_m}}_{second\text{-}order}.$$

The second order term is often omitted with the implicit assumption that is small compared with the first order term [3]. The Total effect is defined as $\alpha_m\lambda_m + \lambda_c$, a summation of the mediated and direct effect. Here, we can take advantage of the properties of variances, namely $\sigma^2_{Tot} = \sigma^2_{\lambda_c} + \sigma^2_{Med} + 2\sigma_{\lambda_c,Med}$, however the Delta-method leads to the same variance estimator. Under mild regularity conditions, (λ_c, λ_m) are normally distributed and independent from α_m, thus $\sigma_{\lambda_c,Med} = \alpha_m\sigma_{\lambda_m\lambda_c}$ leading to a variance estimator for the total effect of $\sigma^2_{Tot} = \sigma^2_{\lambda_c} + \alpha_m^2\sigma^2_{\lambda_m} + \lambda_m^2\sigma^2_{\alpha_m} + 2\alpha_m\sigma_{\lambda_m\lambda_c}$.

On attractive feature of the Delta-method is that it can be extended to multiple mediators or multiple mediating pathways

The DNA copy number aberrations-mRNA-protein-survival pathway

The information stored in the DNA is transcribed to mRNA which in turn is translated to proteins. As a result it would be desired to consider this full pathway however due to technical limitations protein data are rarely available for the researchers. In agreement with the methodology previously described we assume that we modeled the effect of DNA copy number aberrations, mRNA and protein levels on the hazard as

$$\alpha(t\,|\,\mathbf{x}_i) = \beta_0 + \lambda_{m1}Prot + \lambda_{m1}mRNA + \lambda_c DCNA$$

where $\lambda_{m.}$ is the effect of the mediators on the hazard (in our case mRNA levels) while λ_c is the effect of the covariate on the hazard (in our case DNA copy number aberrations). Moreover we assume that levels are explained to a certain degree by DNA copy number aberrations and their relationship can be depicted as

$$mRNA = \alpha_0 + \alpha_m DCNA + \varepsilon_{mRNA}$$

and protein levels are explained to a certain degree by mRNA levels

$$Prot = \gamma_0 + \gamma_m mRNA + \varepsilon_{Prot}.$$

Simplifying these equations leads to

$$\alpha(t\,|\,\mathbf{x}_i) = \beta_0 + DCNA(\alpha_m(\lambda_{mRNA} + \lambda_{Prot}\gamma_m) + \lambda_{DCNA})$$

where $\alpha_m(\lambda_{mRNA} + \lambda_{Prot}\gamma_m)$ is the effect of DNA copy number aberrations on survival status mediated through mRNA and protein, while $\alpha_m(\lambda_{mRNA} + \lambda_{Prot}\gamma_m) + \lambda_{DCNA}$ is the total effect of DNA copy number aberrations on survival status. Applying the Delta-method leads to a variance estimator for the mediated effect of

$$\sigma^2_{med} = \alpha_m^2\sigma^2_{\lambda_{mRNA}} + \left(\lambda_{mRNA} + \lambda_{Prot}\gamma_m\right)^2\sigma^2_{\alpha_m} + \left(\alpha_m\gamma_m\right)^2\sigma^2_{Prot} + \left(\alpha_m\lambda_{Prot}\right)^2\sigma^2_{\gamma_m}.$$

This assumed that mRNA might have an effect on survival that is not mediated through proteins. If we reject this assumption the estimator for the mediator effect simplifies to $\alpha_m \lambda_{Prot} \gamma_m$ and its variance will be

$$\left(\lambda_{Prot}\gamma_m\right)^2 \sigma_{\alpha_m}^2 + \left(\alpha_m \gamma_m\right)^2 \sigma_{Prot}^2 + \left(\alpha_m \lambda_{Prot}\right)^2 \sigma_{\gamma_m}^2 .$$

a. Distribution of the products

The probability distribution function of two random variables X and Y with $U = XY$ is given by $f_V(v) = \int_{-\infty}^{\infty} f_{X,Y}\left(x, \frac{v}{x}\right)\frac{1}{|x|}dx$, which is usually easier to derive than to implement [4]. For two normally distributed random variables the cumulative distribution function is given by

$$F_Z(q) = \iint_A f_{U,V}(u, v \mid \boldsymbol{\mu}, \boldsymbol{\Sigma}) du dv$$

where $U = \frac{X}{\sigma_X}$ and $V = \frac{Y}{\sigma_Y}$ and $\boldsymbol{\mu}, \boldsymbol{\Sigma}$ are the means and the covariance matrix of the two.

This CDF function can be estimated by

$$F_Z(q) = \int_{-\infty}^{\infty} \phi(u - \mu_u)\Phi\left[sign(u) \frac{q/u - \mu_{V|u}}{\sqrt{1-\rho^2}} \right] du$$

where ϕ and Φ are the probability and cumulative distribution functions of the standard normal distribution.

This is readily implemented in the R package 'RMediation' and it is easy to obtain p-values, confidence intervals or other statistics of interest [5].

2. Implementation in R of the Delta-method based inference

The implementation of above detailed estimation procedures is straightforward in R, a versatile programming language. R and its packages contain all the algorithms needed and a simple function written by the first author allows an easy and fast estimation procedure. The user has to make sure that the required packages are preinstalled. Linear regression can be fitted with the function '`lm`' from the base package '`stats`', which is included in the base installation of R. Aalen Additive model can be fitted with the function '`aalen`' from the package '`timereg`' which needs to be installed by the user. All the parameters needed for

estimating the mediation and total effect and their variances can be extracted from the outputs of the two mentioned function.

Confidence Intervals based on the delta method

```
img <- function(Time, Status, Indicator, Covariate, Mediator, method){
  require(timereg)
# Fits Aalen model
out<-
aalen(Surv(Time,Status==Indicator)~const(Covariate)+const(Mediator),max.time=max(Time),n.sim=0
)
Lambda_DNA <- out$gamma[1]
Lambda_RNA <- out$gamma[2]
Var_Lambda_DNA <- out$var.gamma[1,1]
Var_Lambda_RNA <- out$var.gamma[2,2]
Cov_Lambda_DNA_Lambda_RNA <- out$var.gamma[1,2]

fit <- lm(Mediator~Covariate)

Alpha_RNA <- as.numeric(coef(fit)[2])
Var_Alpha_RNA <- as.numeric(vcov(fit)[2,2])

## IE: Indirect effect or mediated effect
## TE: Total effect
## Pm: Relative magnitude
        IE <- Lambda_RNA * Alpha_RNA
        TE <- IE + Lambda_DNA
        Pm <- IE/TE

Var_IE <-  Alpha_RNA^2*Var_Lambda_RNA+ Lambda_RNA^2*Var_Alpha_RNA
Var_TE <-  Var_IE+Var_Lambda_DNA+2*Alpha_RNA*Cov_Lambda_DNA_Lambda_RNA
Var_Pm <- ((IE/TE^2)^2)*Var_Lambda_DNA+
          (((Alpha_RNA*TE-Lambda_RNA*Alpha_RNA^2)/TE^2)^2)*Var_Lambda_RNA+
          (((Lambda_RNA*TE-Lambda_RNA^2*Alpha_RNA)/TE^2)^2)*Var_Alpha_RNA-
          (IE/TE^2)*((Alpha_RNA*TE-Lambda_RNA*Alpha_RNA^2)/TE^2)*Cov_Lambda_DNA_Lambda_RNA

## Results matrix
Return <- matrix(NA, 4, 5)
colnames(Return) <- c('Coef', 'se(Coef)', 'L95%CI', 'U95%CI', 'P-val')
rownames(Return) <- c('Dir Eff', 'Med Eff', 'Tot Eff', 'Pm')

Return[1,1] <- Lambda_DNA; Return[1,2] <- sqrt(Var_Lambda_DNA);
Return[1,3] <- Lambda_DNA + qnorm(0.025)*sqrt(Var_Lambda_DNA)
Return[1,4] <- Lambda_DNA + qnorm(0.975)*sqrt(Var_Lambda_DNA)
Return[1,5] <- 2*pnorm(-abs(Lambda_DNA/sqrt(Var_Lambda_DNA)))

Return[2,1] <- IE; Return[2,2] <- sqrt(Var_IE);
Return[2,3] <- IE + qnorm(0.025)*sqrt(Var_IE)
Return[2,4] <- IE + qnorm(0.975)*sqrt(Var_IE)
Return[2,5] <- 2*pnorm(-abs(IE/sqrt(Var_IE)))

Return[3,1] <- TE; Return[3,2] <- sqrt(Var_TE);
Return[3,3] <- TE + qnorm(0.025)*sqrt(Var_TE)
Return[3,4] <- TE + qnorm(0.975)*sqrt(Var_TE)
Return[3,5] <- 2*pnorm(-abs(TE/sqrt(Var_TE)))

Return[4,1] <- Pm; Return[4,2] <- sqrt(Var_Pm);
Return[4,3] <- Pm + qnorm(0.025)*sqrt(Var_Pm)
Return[4,4] <- Pm + qnorm(0.975)*sqrt(Var_Pm)
Return[4,5] <- ''


class(Return) <- 'numeric'

    return(Return)
 }
```

The provided R script can be used directly; the user only needs to provide the necessary input. First, the user has to define the variable containing the survival time. The second input variable denotes whether the survival time is censored or the individual experienced the outcome (e.g. metastases, death). In survival analysis consensus says that 0 denotes censoring while 1 denotes that the individual experienced the outcome. However, this can vary and we ask the user to define a numeric index denoting the event of interest. Thereafter, the user has to define the covariate and then the mediator.

The line

```
igm(Time, Status, Indicator, Covariate, Mediator)
```

will calculate the desired effects. 'igm' stands for Integrative Genomic Mediation.

References

1. Casella, G. and R.L. Berger, *Statistical Inference, 2nd ed.* 2002.
2. Preacher, K.J., D.D. Rucker, and A.F. Hayes, *Addressing Moderated Mediation Hypotheses: Theory, Methods, and Prescriptions.* Multivariate Behavioral Research, 2007. **42**(1): p. 185-227.
3. Sobel, M.E., *Asymptotic Confidence Intervals for Indirect Effects in Structural Equation Models.* Sociological Methodology, 1982. **13**(ArticleType: research-article / Full publication date: 1982 / Copyright © 1982 John Wiley & Sons): p. 290-312.
4. Glen, A.G., L.M. Leemis, and J.H. Drew, *Computing the distribution of the product of two continuous random variables.* Computational Statistics & Data Analysis, 2004. **44**(3): p. 451-464.
5. Tofighi, D. and D.P. MacKinnon, *RMediation: An R package for mediation analysis confidence intervals.* Behavior Research Methods, 2011. **43**(3): p. 692-700.

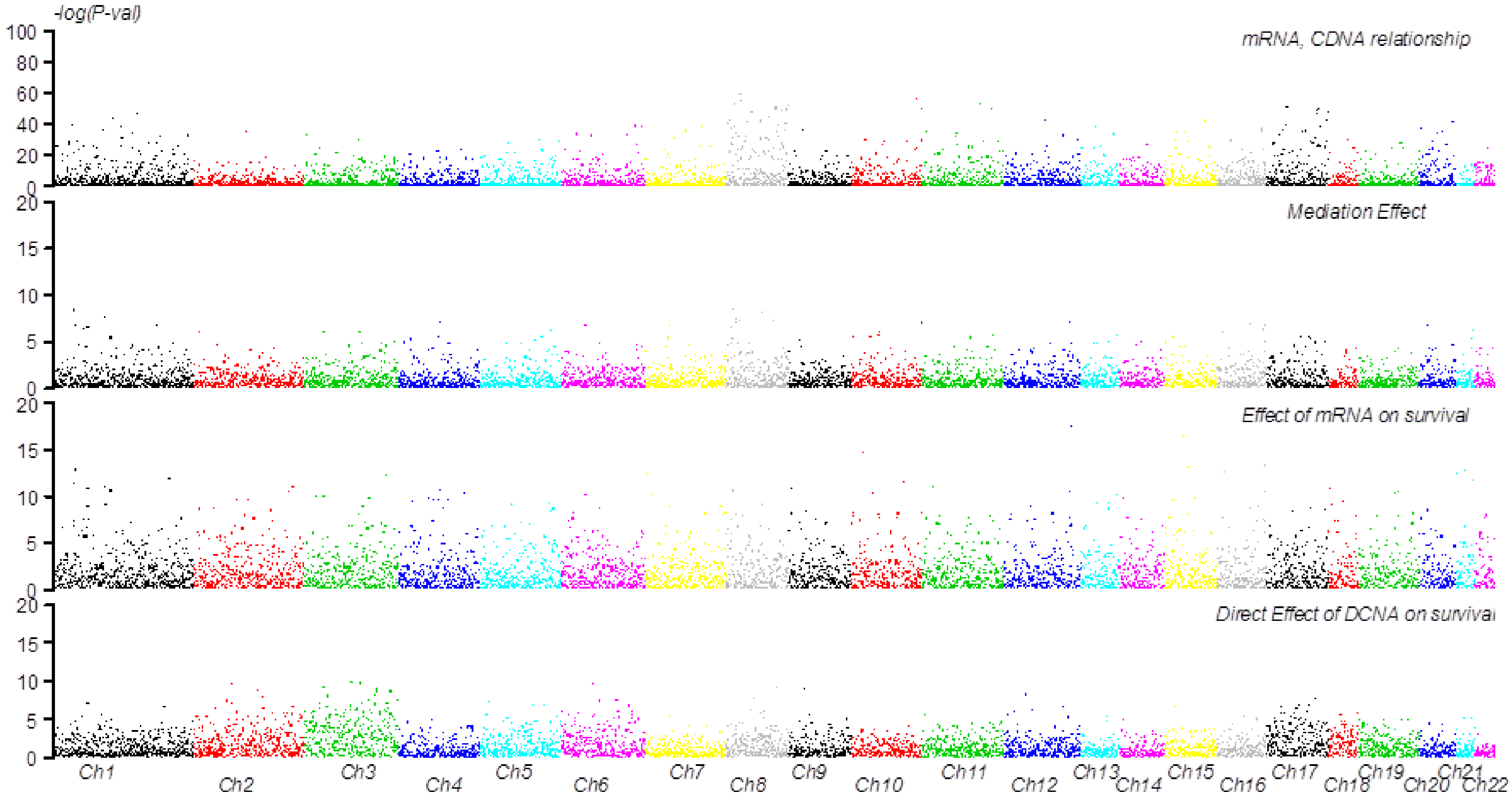

Supplementary figure 1. Manhattan plots for the P-values of (i) slope of the least-squares regression analysis between CDNA-mRNA relationship; (ii) for the significance of the effect of CDNA on survival mediated by mRNA levels; (iii) the effect of mRNA on survival from a prognostic model with CDNA and mRNA levels as predictors and (iv) the effect of CDNA on survival from a prognostic model with CDNA and mRNA levels as predictors.
